# Evaluation of functional genetic variants at 6q25.1 and risk of breast cancer in a Chinese population

**DOI:** 10.1186/s13058-014-0422-x

**Published:** 2014-08-14

**Authors:** Yanru Wang, Yisha He, Zhenzhen Qin, Yue Jiang, Guangfu Jin, Hongxia Ma, Juncheng Dai, Jiaping Chen, Zhibin Hu, Xiaoxiang Guan, Hongbing Shen

**Affiliations:** 1 Department of Medical Oncology, Jinling Hospital, Southern Medical University, 305 East Zhongshan Road, Nanjing, 210000 Jiangsu People's Republic of China; 2 Department of Epidemiology and Biostatistics, Jiangsu Key Laboratory of Cancer Biomarkers, Prevention and Treatment, Cancer Center, School of Public Health, Nanjing Medical University, 818 East Tian-Yuan Road, Nanjing, 211166 Jiangsu People's Republic of China; 3 State Key Laboratory of Reproductive Medicine, Institute of Toxicology, Nanjing Medical University, 818 East Tian-Yuan Road, Nanjing, 211166 People's Republic of China

## Abstract

**Introduction:**

Single-nucleotide polymorphisms (SNPs) at 6q25.1 that are associated with breast cancer susceptibility have been identified in several genome-wide association studies (GWASs). However, the exact causal variants in this region have not been clarified.

**Methods:**

In the present study, we genotyped six potentially functional single-nucleotide polymorphisms (SNPs) within the *CCDC170* and *ESR1* gene regions at 6q25.1 and accessed their associations with risk of breast cancer in a study of 1,064 cases and 1,073 cancer-free controls in Chinese women. The biological function of the risk variant was further evaluated by performing laboratory experiments.

**Results:**

Breast cancer risk was significantly associated with three SNPs located at 6q25.1—rs9383935 in *CCDC170* and rs2228480 and rs3798758 in *ESR1*—with variant allele attributed odds ratios (ORs) of 1.38 (95% confidence interval (CI): 1.20 to 1.57, *P* = 2.21 × 10^-6^), 0.84 (95% CI: 0.72 to 0.98, *P* = 0.025) and 1.19 (95% CI: 1.04 to 1.37, *P* = 0.013), respectively. The functional variant rs9383935 is in high linkage disequilibrium (LD) with GWAS-reported top-hit SNP (rs2046210), but only rs9383935 showed a strong independent effect in conditional regression analysis. The rs9383935 risk allele A showed decreased activity of reporter gene in both the MCF-7 and BT-474 breast cancer cell lines, which might be due to an altered binding capacity of miR-27a to the 3' untranslated region (3' UTR) sequence of *CCDC170*. Real-time quantitative reverse transcription PCR confirmed the correlation between rs9383935 genotypes and *CCDC170* expression levels.

**Conclusions:**

The results of this study suggest that the functional variant rs9383935, located at the 3' UTR of *CCDC170*, may be one candidate of the causal variants at 6q25.1 that modulate the risk of breast cancer.

**Electronic supplementary material:**

The online version of this article (doi:10.1186/s13058-014-0422-x) contains supplementary material, which is available to authorized users.

## Introduction

Breast cancer is the most common female malignancy worldwide, accounting for 23% of all the new cancer cases in 2008 [1]. The incidence of breast cancer is relatively low in China compared with Western countries [2]. However, the incidence has increased rapidly in the past two decades, likely due to the change in lifestyle among Chinese women [2],[3].

Genetic variation has been proved to be a critical factor in discriminating cancer-susceptible individuals [4]. In recent genome-wide association studies (GWASs), researchers have identified numerous single-nucleotide polymorphisms (SNPs) associated with breast cancer risk in diverse populations [5]. Among these SNPs, rs2046210, located between coiled-coil domain containing 170 (*CCDC170*, also called *C6orf97*) and estrogen receptor 1 (*ESR1*) at 6q25.1, was first reported to be associated with the risk of breast cancer in Chinese populations [6]. Subsequently, one intronic variant, rs3757318 in *CCDC170*[7], and another intronic rs9383951 in *ESR1*[8], were also found to be associated with breast cancer risk. To date, investigators in numerous studies have confirmed these associations with breast cancer in this region, especially for rs2046210 [9]-[12].

*ESR1* is a strong candidate susceptibility gene related to breast cancer in the 6q25.1 region (encoding estrogen receptor α), and studies have shown its implication in breast carcinogenesis [13],[14]. Nevertheless, the putative functions of this region are still undefined. Most of the SNPs at 6q25.1 mentioned above have mapped to introns or intergenic regions. In a previous study, a 41-kb block of the 6p25.1 region was systematically analyzed, and significant associations with breast cancer risk were observed for rs1038304, rs6929137, rs2046210 and rs10484919 [15]. However, these variants ae all located upstream of the *ESR1* gene region. Hence, to evaluate the causal variants at 6q25.1 in the development of breast cancer, we screened the potentially functional variants at 6q25.1 within two genes (*ESR1* and *CCDC170*) and assessed their associations with breast cancer risk in a case-control study including 1,064 breast cancer cases and 1,073 controls in Chinese women in Jiangsu province in eastern China. We further evaluated potential biological functions of the SNPs that we found to be associated with breast cancer risk.

## Material and methods

### Study subjects

This study included 1,064 breast cancer cases and 1,073 cancer-free controls, whose characteristics were described previously [16]. Briefly, breast cancer patients were recruited from the First Affiliated Hospital of Nanjing Medical University, the Cancer Hospital of Jiangsu Province and the Gulou Hospital, Nanjing, China, between January 2004 and April 2010. Cases were diagnosed on the basis of standard histological and clinical criteria. The estrogen receptor (ER) and progesterone receptor (PR) status of all breast cancer patients was determined on the basis of immunohistochemistry (IHC) results in pathology reports. The controls were randomly selected from among more than 30,000 participants in a community-based screening program conducted in Jiangsu province, China. The cases and controls were frequency-matched for age (5-year interval). All participants were genetically unrelated. After informed consent was obtained, each individual was interviewed face-to-face to collect information about demographic data and menstrual and reproductive history, and approximately 5 ml of venous blood was collected. This study was approved by the institutional review board of Nanjing Medical University.

### Single-nucleotide polymorphism selection and genotyping

In this study, we applied two approaches to select potential functional SNPs at 6q25.1. First, we focused on those in linkage disequilibrium (LD) with the GWAS-identified SNP rs2046210 at this region and replicated the results in another independent sample. As shown in Additional file 1: Figure S1, a total of 30 SNPs are in LD with rs2046210 (*r*^2^ > 0.8), which were further functionally evaluated by SNPinfo [17] and expression quantitative trait loci (eQTL) analyses [18]. As a result, rs3983935 in *CCDC170* was selected because it is in strong LD with rs2046210 (*r*^2^ = 0.86) and was predicted to (1) affect a potential binding site of microRNA-27a (miR-27a) located in the 3' untranslated region (3' UTR) of *CCDC170* and (2) regulate expression of *CCDC170* in the eQTL analysis.

Using another approach taking into consideration the existence of multiple independent breast cancer susceptibility loci at the 6q25.1 region and the importance of *ESR1* in breast cancer development, we also focused on potential functional SNPs of *ESR1* (chr6:152160379-152466099). Potentially functional SNPs located in the coding (synonymous SNPs, missense SNPs and nonsense SNPs) and regulatory regions (promoter, 5' UTR and 3' UTR) were selected. The SNPs were further filtered according to the LD analysis (*r*^2^ < 0.8) and minor allele frequency (MAF) ≥ 0.05 in Chinese Han population. Six SNPs of *ESR1* met the criteria (Additional file 1: Figure S1), but rs1801132 was excluded because of the failure of probe design. We also included one SNP of *CCDC170* (rs9383935) and five SNPs of *ESR1* (rs488133, rs3798577, rs3798758, rs3798757 and rs2228480). In addition, the well-known SNP at 6q25.1, rs2046210, was selected.

Genomic DNA was isolated from leukocyte pellets of venous blood by proteinase K digestion and followed by phenol-chloroform extraction. All of the DNA samples were checked for quality and quantity with a NanoDrop 2000 spectrophotometer (NanoDrop, Wilmington, DE, USA) and by DNA electrophoresis before genotyping. SNPs were genotyped by using Infinium BeadChip (Illumina, San Diego, CA, USA). The call rate ranged from 97.7% to 97.9% for six SNPs tested in all subjects.

### CCDC170 3' untranslated region luciferase plasmids construct and site-directed mutagenesis

The *CCDC170* 3' UTR containing the rs9383935 G allele was amplified by PCR from human genomic DNA carrying the GG homozygous genotype template with the following primers: sense 5'-AGACGCGTTAAGTCAGGGGCTTTACTAGC-3' and antisense 5'-GCAAGCTTCTGCTGAGTAGTTGGGATTACA-3'. The PCR products were separated in agarose gel, extracted, purified and cloned into the pMIR-REPORT™ miRNA expression reporter vector system (Applied Biosystems, Foster City, CA, USA) with *Mlu*I and *Hin*dIII digestion and then were ligated by T4 DNA ligase to the recombinant constructs (Additional file 2: Figure S2). The plasmid with the rs9383935 G allele was used as the template for the mutation G → A. The site-directed mutagenesis for the plasmid with the A allele construct was generated using a Mut Express Fast Mutagenesis kit (Vazyme Biotech, Nanjing, China). All PCR amplifications and constructs were sequenced to confirm the accuracy of cloning.

### Transient transfection and luciferase assays

MCF-7 and BT-474 cell lines were obtained from Nanjing KeyGen Biotech (Nanjing, China), where they were characterized by mycoplasma detection, DNA fingerprinting, isozyme detection and cell vitality detection. The cell lines were maintained in growth medium supplemented with 10% heat-inactivated fetal bovine serum and 100 U/ml penicillin and 100 μg/ml streptomycin in a 37°C incubator supplemented with 5% CO_2_ (MCF-7 with Dulbecco's modified Eagle's medium and BT474 with RPMI 1640 medium). Cell lines were seeded into 24-well culture plates and incubated for 24 hours before transfection. Transfections were performed using Lipofectamine 2000 reagent (Invitrogen, Carlsbad, CA, USA) according to the manufacturer's protocol. The luciferase plasmids (empty vector for control and vectors with different rs9383935 alleles) were cotransfected, respectively, into different cells with synthesized mature miR-27a-3p mimic (5'-UUCACAGUGGCUAAGUUCCGC-3') or miRNA negative control. The pRL-SV40 plasmid (Promega, Madison, WI, USA) was also cotransfected as an internal control. After a 24-hour incubation, Firefly and *Renilla* luciferase activities were determined with the Dual-Luciferase Reporter Assay System (Promega) on a luminometer (BioTek, Winooski, VT, USA). Three independent experiments with six replicates were performed in triplicates.

### Transfection of has-miR-27a-3p in MCF-7 breast cancer cell line

The has-miR-27a mimic and the negative control RNA duplex were transfected into MCF-7 cells seeded in six-well plates using Lipofectamine 2000 reagent. Cells were harvested 16 hours after transfection, and RNAs were isolated. Two independent transfection experiments were conducted in triplicate. Real-time PCR analysis of mRNA levels was performed as well.

### Real-time quantitative reverse transcription PCR of CCDC170 and ESR1

Total RNAs from peripheral blood samples of 122 healthy individuals or breast cancer cell lines were extracted using TRIzol reagent (Invitrogen) according to the manufacturer's instructions. RNAs were reverse-transcribed into cDNA using PrimeScript™ RT Master Mix (TaKaRa Bio, Tokyo, Japan). Real-time quantitative reverse transcription PCR (qRT-PCR) was carried out with a TaqMan Gene Expression Assay (Applied Biosystems) contained probes for *CCDC170* (Hs00228128_m1) and *ESR1* (Hs00174860_m1). Each assay was analyzed in triplicate, and *ACTB* (Hs99999903_m1) was used as an endogenous control. The threshold cycle (C_t_) was determined for each assay. The relative expression levels were calculated using the 2^−ΔCt^ method.

### Statistical analysis

Differences in demographic characteristics, selected variables and frequencies of alleles and genotypes between the cases and the controls were analyzed by using Student's *t*-test (for continuous variables) and χ^2^ test (for categorical variables). The Hardy-Weinberg equilibrium (HWE) for the genotype distribution of each SNP was evaluated using the goodness-of-fit χ^2^ test by comparing the observed genotype frequencies with the expected ones among the controls. Logistic regression analyses were employed to evaluate the associations between SNPs and the risk of breast cancer by estimating the odds ratios (ORs) and their 95% confidence intervals (CIs) with adjustment for potential confounders such as age, age at menarche and menopausal status. The heterogeneity of associations between subgroups was assessed using the χ^2^-based Q-test. Differences in measurements of luciferase assays and miR-27a-3p transfection experiments between subgroups were examined using the *t*-test. Differences in the expression levels of *CCDC170* and *ESR1* among GG, GA and AA genotypes of rs9383935 were assessed by nonparametric trend test. All of the statistical analyses were two-sided with *P* < 0.05 taken as the significance level, and they were performed with SAS 9.1.3 software (SAS Institute, Cary, NC, USA).

## Results

The characteristics of the 1,064 breast cancer cases and the 1,073 cancer-free controls have been presented elsewhere [16]. In brief, age variable was comparable between cases and controls (*P* > 0.05). The breast cancer cases showed an earlier age at menarche, a later age at first live birth and a lower proportion of natural postmenopausal status compared with the controls (all *P* < 0.05). Among the 869 patients with immunohistochemistry records for tumor tissues, 490 cases (56.4%) were ER-positive and 506 cases (58.2%) were PR-positive.

The genotype distributions of the seven SNPs between cases and controls and their associations with breast cancer risk are summarized in Table 1. The observed genotype frequencies of seven SNPs followed Hardy-Weinberg equilibrium among the controls (*P* > 0.05 for all seven SNPs). Logistic regression analysis revealed that the minor rs9383935 A allele of *CCDC170* was significantly associated with an increased risk of breast cancer in an additive model (per-allele OR = 1.38, 95% CI: 1.20 to 1.57, *P* = 2.21 × 10^-6^). A similar association was observed for the rs2046210 A allele (per-allele OR = 1.32, 95% CI: 1.16 to 1.50, *P* = 2.04 × 10^-5^). In addition, the rs3798758 A allele of *ESR1* was associated with an increased risk of breast cancer (per-allele OR = 1.19, 95% CI: 1.04 to 1.37, *P* = 0.013), whereas the rs2228480 A allele of *ESR1* was associated with a decreased risk (per-allele OR = 0.84, 95% CI: 0.72 to 0.98, *P* = 0.025). However, no significant associations were observed for rs488133, rs3798577 or rs3798757. After correction for multiple testing (*n* = 7), rs9383935 and rs2046210 of *CCDC170* were still significantly associated with breast cancer risk (*P* < 0.007).

**Table 1:** **Summary of associations between seven single-nucleotide polymorphisms at 6q25.1 and breast cancer risk** ^**a**^

SNP	Position (hg18)	Associated gene	Location	Allele^b^	Cases^c^(***n***= 1064)	Controls^c^(***n*** = 1073)	MAF^d^	HWE^e^	Additive model^f^	
									OR (95% CI)	***P***-value
rs9383935	151981541	*CCDC170*	3' UTR	G/A	414/500/149	528/439/106	0.38/0.30	0.31	1.38 (1.20 to 1.57)	2.21 × 10^-6^
rs2046210	151990059	Intergenic	-	G/A	349/517/196	447/475/150	0.43/0.36	0.19	1.32 (1.16 to 1.50)	2.04 × 10^-5^
rs488133	152167137	*ESR1*	intron	G/A	867/184/11	906/160/7	0.10/0.08	1.00	1.15 (0.93 to 1.43)	0.208
rs2228480	152461788	*ESR1*	Thr594Thr	G/A	682/340/40	633/387/49	0.20/0.23	0.34	0.84 (0.72 to 0.98)	0.025
rs3798577	152462823	*ESR1*	3' UTR	A/G	336/527/196	305/541/226	0.43/0.46	0.67	0.90 (0.79 to 1.02)	0.093
rs3798758	152463547	*ESR1*	3' UTR	C/A	520/441/98	577/425/69	0.30/0.26	0.48	1.19 (1.04 to 1.37)	0.013
rs3798757	152465936	*ESR1*	3' UTR	A/G	856/185/20	879/182/10	0.11/0.09	0.86	1.16 (0.95 to 1.43)	0.149

^a^CI, Confidence interval; OR, Odds ratio; SNP, Single-nucleotide polymorphism. ^b^Major/minor allele. ^c^Major homozygote/heterozygote/rare homozygote between cases and controls. ^d^Minor allele frequency (MAF) among cases/controls. ^e^ Hardy-Weinberg equilibrium (HWE) test among controls. ^f^Logistic regression with adjustment for age, age at menarche and menopausal status.

We further evaluated the associations of rs9383935, rs2046210, rs3798758 and rs2228480 with risk of breast cancer by subgroups of age, age at menarche and first live birth, menopausal status (premenopausal and natural menopausal) and subtype of breast cancer (ER and PR status). As shown in Table 2, the associations for rs9383935 and rs2046210 were significant in all the subgroups (all *P* < 0.05). Specifically, the association with rs3798758 was significant among women of younger age, older age at menarche and premenopausal status (*P* = 0.005, 0.022 and 0.037, respectively). For rs2228480, a significant association was also observed in women of an older age at both menarche and birth of first child (*P* = 0.005 and *P* = 0.048, respectively). In subtypes of breast cancer, rs3798758 was significantly associated with risk of ER-positive breast cancers (per-allele OR = 1.21, 95% CI: 1.02 to 1.48, *P* = 0.030). Meanwhile, the rs2228480 A allele showed a protective effect regardless of ER and/or PR status. However, no heterogeneity was observed in any strata of the subgroups.

**Table 2:** **Stratified analysis of the associations between rs9383935, rs2046210, rs2228480 and rs3798758 with breast cancer risk**

Characteristics	rs9383935		***P***-value^b^	rs2046210		***P***-value^b^	rs2228480		***P***-value^b^	rs3798758		***P***-value^b^
	OR (95% CI)^a^	***P***-value^a^		OR (95% CI)^a^	***P***-value^a^		OR (95% CI)^a^	***P***-value^a^		OR (95% CI)^a^	***P***-value^a^	
Age, yr												
<51	1.43 (1.19 to 1.71)	0.0001	0.556	1.36 (1.15 to 1.62)	0.0005	0.599	0.82 (0.67 to 1.02)	0.071	0.798	1.31 (1.08 to 1.57)	0.005	0.138
≥51	1.32 (1.09 to 1.60)	0.0048		1.27 (1.06 to 1.53)	0.0114		0.86 (0.68 to 1.07)	0.180		1.06 (0.86 to 1.30)	0.613	
Age at menarche, yr												
<16	1.39 (1.15 to 1.68)	0.0006	0.914	1.31 (1.09 to 1.57)	0.0039	0.879	0.98 (0.79 to 1.22)	0.849	0.061	1.13 (0.93 to 1.38)	0.234	0.466
≥16	1.37 (1.14 to 1.65)	0.0007		1.34 (1.12 to 1.59)	0.0012		0.73 (0.59 to 0.91)	0.005		1.25 (1.03 to 1.52)	0.022	
Age at first live birth, yr												
<24	1.52 (1.18 to 1.95)	0.0011	0.368	1.39 (1.09 to 1.77)	0.0077	0.621	0.91 (0.68 to 1.21)	0.505	0.611	1.21 (0.93 to 1.58)	0.152	0.730
≥24	1.32 (1.13 to 1.56)	0.0007		1.29 (1.11 to 1.51)	0.0012		0.83 (0.69 to 1.00)	0.048		1.15 (0.97 to 1.36)	0.114	
Menopausal status												
Premenopausal	1.37 (1.14 to 1.65)	0.0010	0.876	1.30 (1.08 to 1.55)	0.0049	0.725	0.83 (0.66 to 1.04)	0.109	0.889	1.23 (1.01 to 1.49)	0.037	0.300
Postmenopausal^c^	1.40 (1.15 to 1.70)	0.0008		1.36 (1.13 to 1.64)	0.0014		0.85 (0.68 to 1.06)	0.151		1.06 (0.86 to 1.30)	0.613	
ER status												
Positive	1.30 (1.10 to 1.53)	0.0018	0.324	1.24 (1.06 to 1.46)	0.0068	0.148	0.80 (0.65 to 0.97)	0.025	0.765	1.21 (1.02 to 1.48)	0.030	0.732
Negative	1.47 (1.23 to 1.75)	2.5 × 10^-5^		1.48 (1.24 to 1.76)	1.05 × 10^-5^		0.76 (0.61 to 0.95)	0.014		1.16 (0.96 to 1.40)	0.126	
PR status												
Positive	1.33 (1.13 to 1.57)	0.0005	0.618	1.31 (1.12 to 1.53)	0.0008	0.645	0.79 (0.65 to 0.96)	0.020	0.886	1.18 (0.99 to 1.41)	0.063	0.869
Negative	1.42 (1.19 to 1.70)	0.0001		1.38 (1.16 to 1.64)	0.0003		0.77 (0.62 to 0.97)	0.023		1.21 (1.00 to 1.46)	0.055	

^a^Adjusted for age, age at menarche and menopausal status where appropriate. ^b^*P*-value for heterogeneity. ^c^Postmenopausal status for natural menopause.

To substantiate these findings, we performed logistic regression analyses conditioned on the four significant SNPs at 6q25.1 (Table 3). We first focused on the two SNPs, rs9383935 and rs2046210, which were in LD (*r*^2^ = 0.86) and passed multiple testing correction. After adjusting for rs2046210, the data for rs9383935 remained significant (*P* = 0.025). In the reverse condition, the results derived for rs2046210 showed a much weaker association (*P* = 0.810). Next, we included all four SNPs in the same model and found that the effects of rs2228480 and rs3798758 were apparently decreased after conditioned on the three other SNPs (*P* = 0.069 and *P* = 0.082, respectively), as was the result for rs2046210 (*P* = 0.901); however, the effect of rs9383935 remained significant (*P* = 0.019). Taken together, the results of the conditional regression analysis indicated a strong independent effect of rs9383935 on breast cancer risk in our study population.

**Table 3:** **Conditional regression analysis of rs9383935, rs2046210, rs2228480 and rs3798758**

SNP	Adjusted for rs9383935		Adjusted for rs2046210		Adjusted for rs2228480		Adjusted for rs3798578	
	OR (95% CI)^a^	***P***-value^a^	OR (95% CI)^a^	***P***-value^a^	OR (95% CI^a^	***P***-value^a^	OR (95% CI)^a^	***P***-value^a^
rs9383935	1.37 (1.05 to 1.77)^b^	0.019^b^	1.34 (1.04 to 1.74)	0.025	1.38 (1.21 to 1.58)	1.62 × 10^-6^	1.38 (1.21 to 1.57)	2.11 × 10^-6^
rs2046210	1.03 (0.80 to 1.32)	0.810	1.02 (0.79 to 1.31)^b^	0.901^b^	1.32 (1.16 to 1.50)	1.83 × 10^-5^	1.31 (1.16 to 1.49)	2.72 × 10^-5^
rs2228480	0.83 (0.71 to 0.97)	0.018	0.83 (0.71 to 0.97)	0.020	0.86 (0.73 to 1.01)^b^	0.069^b^	0.87 (0.74 to 1.03)	0.097
rs3798758	1.19 (1.03 to 1.37)	0.016	1.19 (1.03 to 1.37)	0.016	1.15 (0.99 to 1.33)	0.062	1.14 (0.98 to 1.32)^b^	0.082^b^

^a^Adjusted for age, age at menarche, menopausal status where appropriate. ^b^Logistic regression analysis conditioned on the three other single-nucleotide polymorphisms (SNPs).

**Figure 1:**
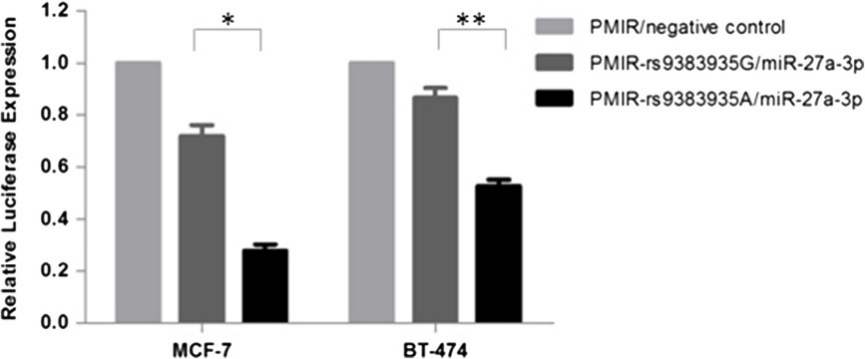
**Luciferase reporter assays of rs9383935 G/A in MCF-7 and BT-474 cell lines.** The G allele constructs had significantly higher luciferase activity than the plasmids bearing the A allele in both the MCF-7 (*P = 0.012) and BT-474 (**P = 0.018) cell lines. Shown are the mean ± SD of relative luciferase expression for plasmids with different alleles after normalized by control groups in parallel experiments.

**Figure 2:**
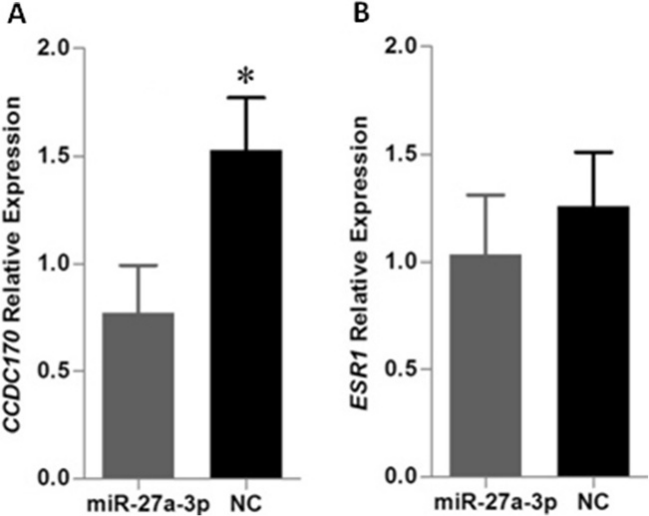
**Transfection of miR-27a-3p in MCF-7 breast cancer cell line. (A)** miR-27a-3p downregulated *CCDC170* mRNA expression after 16-hour transfection compared with negative control (NC) (*P* = 0.016). **(B)** *ESR1* mRNA expression was not significantly different between the two groups (*P* = 0.361).

As predicted with RNAhybrid, miR-27a-3p has a lower minimum free energy (MFE) with the G allele (|MFE| = 21.5 kcal/mol) of rs9383935 in *CCDC170* than that with the A allele (|MFE| = 25.1 kcal/mol) (Additional file 3: Figure S3). Thus, we proposed that the A allele may decrease the expression of *CCDC170*, possibly by reducing miRNA repression. Therefore, we constructed the plasmids containing the rs9383935 G or A allele to determine whether this variant could affect gene expression. When we cotransfected miR-27a-3p mimic into MCF-7 cell line, we found that the activity of the reporter gene with the rs9383935 A allele was significantly decreased compared with that of the G allele (0.72 versus 0.28; *P* = 0.012). Similar effects were observed in the BT-474 cell line (0.87 versus 0.53, *P* = 0.018) (Figure 1). Subsequently, we transfected miR-27a-3p into MCF-7 breast cancer cells and validated its regulation of the endogenous *CCDC170*. The qRT-PCR assay showed that miR-27a-2p significantly decreased *CCDC170* mRNA level compared with the negative controls (*P* = 0.016, Figure 2A); however, we did not find significant changes in *ESR1* expression after miR-27a-3p transcription (*P* = 0.361) (Figure 2B).

**Figure 3:**
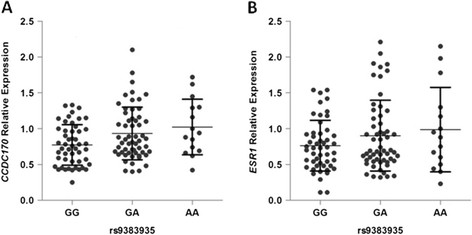
**Correlation of rs9383935 genotypes with** ***CCDC170*** **and** ***ESR1*** **relative expression. (A)** *P* = 0.012. **(B)** *P* = 0.238.

To further analyze the influence of rs9383935 on gene expression, we examined *CCDC170* and *ESR1* expression in 122 healthy individuals using qRT-PCR. We found that the subjects with the risk AA genotype had the highest level of *CCDC170* expression, followed by subjects with the GA and GG genotypes (*P* = 0.012) (Figure 3A). However, we did not detect a difference in *ESR1* expression between different rs9383935 genotypes (*P* = 0.238) (Figure 3B).

## Discussion

The 6q25.1 region was first identified as a breast cancer susceptibility locus in a GWAS of Chinese women [6], which was well-replicated in subsequent follow-up studies [12]. Rs2046210 is an intergenic variant located 29 kb upstream of *ESR1* 5' UTR and 6 kb downstream of *CCDC170* 3' UTR. eQTL analysis indicated that rs2046210 was significantly associated with *CCDC170* expression [19]. However, it has been suggested that there is no strong evidence to support a role of SNP rs2046210 as a functional variant regulating the expression of *CCDC170* according to the results of bioinformatics analysis from SNPinfo and RegulomeDB [20]. Nevertheless, these findings suggest that other genetic variants highly correlated with rs2046210 at 6q25.1 may be functional and may modulate individual susceptibility to breast cancer.

Recently, Cai *et al.* conducted luciferase assays with four fragments from the 36-kb region harboring rs2046210, and they found that rs6913578, in strong LD with rs2046210 (*r*^2^ > 0.8 in CHB and European ancestry (CEU)), might be a functional variant acting as a regulator of enhancement [21], suggesting that genetic variants at 6q25.1 may function through modulating a transcriptional mechanism. In contrast, in the present study, we analyzed functional SNPs in strong LD (*r*^2^ > 0.8) with rs2046210, especially for those SNPs in the miRNA binding sites. Interestingly, we found that rs9383935 highly correlated with rs2046210 was significantly associated breast cancer susceptibility, which result from the allele's differential binding affinity of miR-27a in the 3' UTR of *CCDC170* as evaluated by luciferase assays and miRNA transfection in breast cancer cell lines. Notably, this functional variant in *CCDC170* showed a strong independent effect, even when well-known rs2046210 was adjusted for in the logistic regression analysis. Moreover, qRT-PCR results confirmed that the eQTL annotation linked rs9383935 to *CCDC170* expression. *CCDC170* is an uncharacterized open reading frame (ORF) located upstream of *ESR1*. Several genetic variants within this gene have been implicated in GWASs as being associated with the risk of breast cancer and bone mineral density [7],[22]-[24]. For example, Dunbiers *et al*. observed that three ORFs including *CCDC170* expression were highly correlated with *ESR1* in aromatase inhibitor-treated breast tumor and breast cancer cell lines [25]. miR-27a is known to function as an onco-miRNA in several cancer cell lines [26]-[28] and to play an important part in cell differentiation and proliferation [28],[29]. Furthermore, overexpression of miR-27a could promote epithelial-mesenchymal transition in cancer metastasis and could be a promising prognostic factor in breast cancer [30].

Taken together, these findings suggest that the variant rs9383935 may modulate individual susceptibility to breast cancer, possibly through regulating miR-27a inhibition of *CCDC170* expression. On the basis of the above results, rs9383935 could be considered a potential causal variant at 6q25.1. Additional studies are needed to validate our findings and extend the role of *CCDC170* in the etiology of breast cancers.

Researchers in numerous studies have evaluated the associations between variants in *ESR1* and breast cancer risk [31],[32]. As mediated by estrogen, ERα can directly bind to estrogen response elements or indirectly interacts with chromatin through tethering to other transcription factors, such as coactivators or corepressors [13]. Clinical and epidemiological studies have shown that ERα affects cancer initiation, progression and response to treatment, especially in breast cancer [14]. In a previous study, we conducted a meta-analysis in diverse populations and provided evidence for associations between two SNPs (rs2234693, rs1801132) of *ESR1*and breast cancer susceptibility [31]. In a follow-up study, we performed a case°Control study with 878 cases and 900 controls on rs2234693 and rs1801132 validated the association of rs2234693 with breast cancer risk in Chinese women (OR = 0.85, 95% CI: 0.74 to 0.98, *P* = 0.024), but not for rs1801132 [15].

As an extension of our previous studies, in the present study we evaluated potentially functional SNPs of *ESR1* in a larger sample size comprising 1,064 cases and 1,073 controls. We found significant associations of rs3798758 and rs2228480 with breast cancer risk Chinese women. The SNP rs2228480, located in exon 8 of *ESR1*, is a synonymous variant. The exon 8 involves in the assembling of the C-terminal region of ERα [33]. This region modulates interaction between ERα and other transcription factors, likely to change the affinity of ERα with DNA [33]. Although synonymous variants do not change amino acid sequences, accumulated evidence indicates that these variants can modify mRNA splicing, stability, structure and translation process [34]. For example, Nackley *et al*. showed that diverse mRNA secondary structures with different stability were correlated to protein expression levels [35]. Another SNP, rs3798758, may also influence miRNA binding, including miR-383, a negative regulator of proliferation [36]. Nevertheless, in the present study, both of these two SNPs showed weak effects in our multivariate logistic regression analysis, which failed to show their independent associations with breast cancer risk. However, some variants in intron regions which might be involved in alternative splicing or enhancer manipulation were not included in the present study. Additional investigations focused on these regions are warranted to expand the understanding of 6q25.1 in breast cancer susceptibility.

## Conclusions

Overall, in the present study, we evaluated six potentially functional SNPs within the 6q25.1 region and confirmed that rs9383935, rs3798758 and rs2228480 were associated with breast cancer in Chinese women, and we also replicated rs2046210 in accordance with previous reports. Specifically, the *CCDC170* rs9383935 showed the most prominent effect of any other variants of *ESR1* or rs2046210, which provides new evidence for the role of the 6q25.1 region in breast cancer susceptibility. Further functional investigations of *CCDC170* and 6q25.1 are warranted to fully reveal the mechanisms underlying the observed association with risk of breast cancer.

## Authors' contributions

HS, XG and ZH conceived of the study and managed the overall project. YW, YH, ZQ, YJ and JC were responsible for sample-processing and performed the experiments. YW and GJ interpreted the initial statistical analyses and drafted the manuscript. JD and YW performed data management and checked the statistical analyses. HS, ZH, XG, GJ and HM contributed to the editing of the manuscripts. All authors read and approved the final manuscript.

## Additional files

## Electronic supplementary material

Additional file 1: Figure S1.: Overview of the 6q25.1 region (chr6:151767683-152466099) from the UCSC Genome Browser (NCBI36/hg18). The upper panel shows the 698.4-kb region in 6q25.1 contain four genes: *RMND1*, *C6orf211*, *CCDC170* and *ESR1*. The lower panel shows linkage disequilibrium (LD) plots of 31 SNPs in LD with rs2046210 and 6 selected functional SNPs marked with an asterisk. LD values between SNPs as indicated in the diamonds were measured by *r*^2^ in Chinese descent (CHB). For example, the *r*^2^ value between rs2046210 and rs9383935 was 0.86 in CHB. (JPEG 665 KB)

Additional file 2: Figure S2.: The construction of *CCDC170* 3' UTR luciferase reporter plasmid. (JPEG 219 KB)

Additional file 3: Figure S3.: The predicted binding affinity of miR-27a-3p and *CCDC170* 3' UTR. The figures and the values of minimum free energy (MFE) were generated in RNAhybrid (http://bibiserv.techfak.uni-bielefeld.de/rnahybrid/). Different alleles of rs9383935 are marked with squares. (JPEG 102 KB)

Below are the links to the authors’ original submitted files for images.

Authors’ original file for figure 1

Authors’ original file for figure 2

Authors’ original file for figure 3

## Abbreviations

CCDC170: Coiled-coil domain containing 170
CI: Confidence interval
eQTL: Expression quantitative trait loci
ER: Estrogen receptor
ESR1: Estrogen receptor 1
GWAS: Genome-wide association study
HWE: Hardy–Weinberg equilibrium
IHC: Immunohistochemistry
LD: Linkage disequilibrium
MAF: Minor allele frequency
MFE: Minimum free energy
OR: Odds ratio
PR: Progesterone receptor
SNP: Single-nucleotide polymorphism
UTR: Untranslated region
