# Supplementary figures and images for: Evaluation of functional genetic variants at 6q25.1 and risk of breast cancer in a Chinese population
Source: Breast Cancer Res. 2014 Aug 14;16:422. doi: 10.1186/s13058-014-0422-x (PMC4303231; doi:10.1186/s13058-014-0422-x)

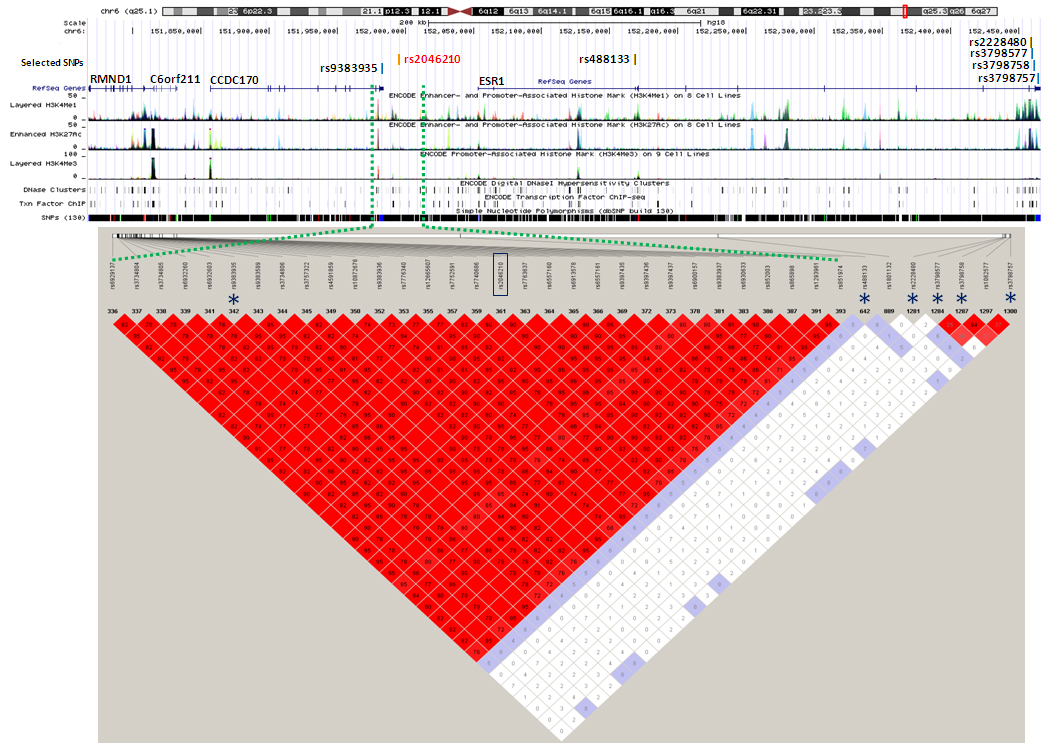

Supplement: Supplementary file 1 — Additional file 1: Figure S1.: Overview of the 6q25.1 region (chr6:151767683-152466099) from the UCSC Genome Browser (NCBI36/hg18). The upper panel shows the 698.4-kb region in 6q25.1 contain four genes: RMND1, C6orf211, CCDC170 and ESR1. The lower panel shows linkage disequilibrium (LD) plots of 31 SNPs in LD with rs2046210 and 6 selected functional SNPs marked with an asterisk. LD values between SNPs as indicated in the diamonds were measured by r2 in Chinese descent (CHB). For example, the r2 value between rs2046210 and rs9383935 was 0.86 in CHB. (JPEG 665 KB) [file 13058_2014_422_MOESM1_ESM.jpeg]

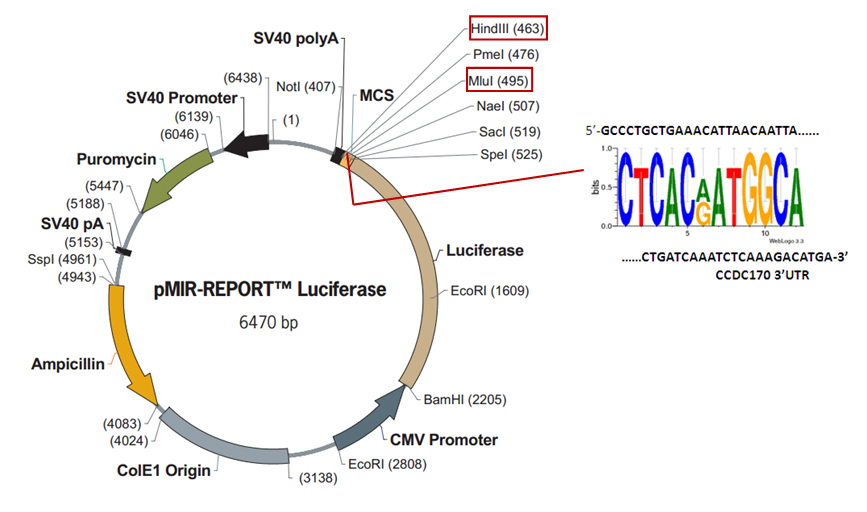

Supplement: Supplementary file 2 — Additional file 2: Figure S2.: The construction of CCDC170 3' UTR luciferase reporter plasmid. (JPEG 219 KB) [file 13058_2014_422_MOESM2_ESM.jpeg]

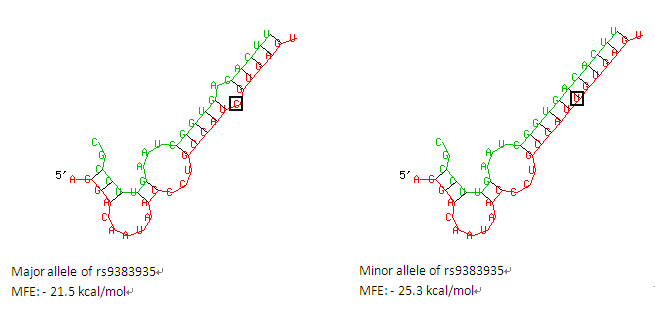

Supplement: Supplementary file 3 — Additional file 3: Figure S3.: The predicted binding affinity of miR-27a-3p and CCDC170 3' UTR. The figures and the values of minimum free energy (MFE) were generated in RNAhybrid (http://bibiserv.techfak.uni-bielefeld.de/rnahybrid/). Different alleles of rs9383935 are marked with squares. (JPEG 102 KB) [file 13058_2014_422_MOESM3_ESM.jpeg]

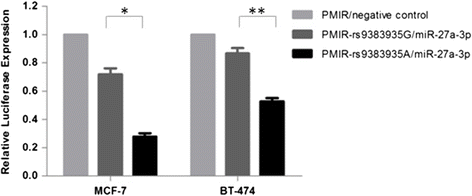

Supplement: Supplementary file 4 — Authors’ original file for figure 1 [file 13058_2014_422_MOESM4_ESM.gif]

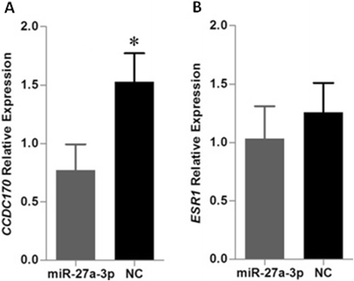

Supplement: Supplementary file 5 — Authors’ original file for figure 2 [file 13058_2014_422_MOESM5_ESM.gif]

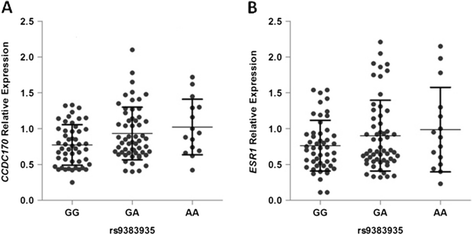

Supplement: Supplementary file 6 — Authors’ original file for figure 3 [file 13058_2014_422_MOESM6_ESM.gif]
